# Harmonization of Zika neutralization assays by using the WHO International Standard for anti-Zika virus antibody

**DOI:** 10.1038/s41541-019-0135-3

**Published:** 2019-10-14

**Authors:** Giada Mattiuzzo, Ivana Knezevic, Mark Hassall, James Ashall, Sophie Myhill, Valwynne Faulkner, Jason Hockley, Peter Rigsby, Dianna E. Wilkinson, Mark Page, the collaborative study participants, Marco Donolato, Sally Baylis, Constanza Yue, Fabian Elgner, In-Kyu Yoon, Jae Seung Yang, Manki Song, Gabriel Gonzalez-Escobar, Richard Brindle, Richard Tedder, Steve Dicks, Ines Ushiro-Lumb, Sarah Williams-McDonald, Sanjai Kumar, Keith Peden, Hana Golding, Surender Khurana, Matthew Bonaparte, Anna Durbin, Hansi Dean, Stephanie Sonnberg, Wayne Hogrefe, James Crowe, Thomas Voss, Matthew Collins, Theodore Pierson, Katherine Burgomaster, Kimberly Dowd, Louise Sigismondi, Dhammika Gunasekera, James Wassenberg, Kenneth Eckels, Rafael De La Barrera

**Affiliations:** 1 Division of Virology, National Institute for Biological Standards and Control (NIBSC), Blanche Lane, South Mimms, Potters Bar, Hertfordshire, EN6 3QG UK; 2 Department of Essential Medicines and Health Products, World Health Organization, Avenue Appia 20, 1211 Geneva, Switzerland; 3 Department of Biostatistics, National Institute for Biological Standards and Control (NIBSC), Blanche Lane, South Mimms, Potters Bar, Hertfordshire, EN6 3QG UK; 4 BlueSense Diagnostics ApS, Copenhagen, Denmark; 5 Paul-Ehrlich-Institut, Langen, Germany; 6 International Vaccine Institute, Seoul, Korea; 7 Caribbean Public Health Agency, St. Clair- Port of Spain, Trinidad and Tobago; 8 Virus Reference Department, Public Health England, London, UK; 9 Center for Biologics Evaluation and Research, US Food and Drug Administration, Bethesda, MD USA; 10 Sanofi Pasteur, Swiftwater, PA USA; 11 Johns Hopkins Bloomberg School of Public Health, Baltimore, MD USA; 12 Takeda Vaccines Inc., Deerfield, IL USA; 13 Q2 Solutions-Vaccines, Focus Diagnostics Inc., San Juan Capistrano, CA USA; 14 Vanderbilt University Medical Center, Nashville, TN USA; 15 University of North Carolina, Chapel Hill, NC USA; 16 National Institute of Allergy and Infectious Diseases, National Institutes of Health, Bethesda, MD USA; 17 Chembio Diagnostic Systems Inc., Medford, NY USA; 18 DiaSorin Inc., Stillwater, MN USA; 19 Walter Reed Army Institute of Research, Silver Spring, MD USA

**Keywords:** Policy and public health in microbiology, Viral infection

## Abstract

During outbreaks of emerging viruses, such as the Zika outbreak in 2015–2016, speed and accuracy in detection of infection are critical factors to control the spread of the disease; often serological and diagnostic methods for emerging viruses are not well developed and validated. Thus, vaccines and treatments are difficult to evaluate due to the lack of comparable methods. In this study, we show how the 1st WHO International Standard for anti-Zika antibody was able to harmonize the neutralization titres of a panel of serological Zika-positive samples from laboratories worldwide. Expression of the titres in International Unit per millilitre reduced the inter-laboratory variance, allowing for greater comparability between laboratories. We advocate the use of the International Standard for anti-Zika virus antibodies for the calibration of neutralization assays to create a common language, which will permit a clear evaluation of the results of different clinical trials and expedite the vaccine/treatment development.

## Zika virus: Harmonizing multisite neutralization assays

Protocols for achieving fast and effective detection of Zika virus (ZIKV) infections during outbreaks are limited by the cross-reactivity of immunoassays and the inter-laboratory variability of results, which may adversely affect response planning in the field and slow the development of new diagnostic tools and therapies. Giada Mattiuzzo and colleagues now applied the WHO International Standard for ZIKV-specific antibodies to harmonize the neutralization titres generated from a panel of serological ZIKV-positive samples analysed in multiple laboratories across the world. The use of the International Standard, which includes a standard sample (a defined pool of sera from confirmed ZIKV-positive cases) and an agreed International Unit, reduced the inter-laboratory variance in neutralization assay results performed on ZIKV of Asian lineage, and enabled a better comparison and integration of the multisite data. This work establishes the first International Standard for an Asian lineage ZIKV-specific antibody for facilitating ZIKV diagnostics, prevention and treatment.

## Introduction

Zika virus (ZIKV) is a flavivirus mainly transmitted by *Aedes* species mosquitoes. ZIKV was discovered in 1947 and was only known as a cause of sporadic mild disease in Africa and Asia.^1,2^ The first major outbreak occurred in 2007 in Yap Island with an attack rate as high as 70% of the population.^3^ In 2013, during an outbreak in French Polynesia with a similarly high attack rate, the possible association with Guillain–Barré Syndrome was uncovered.^4^ The outbreak of ZIKV in the Latin American region in 2015–2016 was the largest recorded epidemic of Zika disease as of early 2019. Although the majority of Zika infections are asymptomatic or result in mild symptoms, the large number of cases in Latin America provided evidence for a correlation between Zika infection and increased cases of microcephaly and other neurological diseases.^5,6^ This association prompted the World Health Organization (WHO) to declare the outbreak a Public Health Emergency of International Concern (PHEIC) in February 2016.^7^ WHO called on the global research and product development (R&D) communities to prioritize the development of vaccines together with improved diagnostics, and innovative vector control strategies for ZIKV R&D. Although the PHEIC was declared over by the WHO Director-General in November 2016, ZIKV remains an enduring public health challenge requiring continued action, as new outbreaks may occur.^8^ Many uncertainties remain with regard to disease epidemiology and transmission, and, therefore, projecting the future evolution of the ZIKV epidemic and further spread based on the current knowledge remains difficult. In February 2018, WHO published “Zika Vaccine Development Technology Roadmap” to support development of a vaccine for outbreak use with the characteristics proposed within the Target Product Profile.^9,10^ In that context, standardization of virologic and immunologic assays for ZIKV vaccine development was identified as one of the priority areas. There is no licensed vaccine or treatment available, but development of these products is ongoing. According to the current knowledge on the transmission of ZIKV and experiences with past disease outbreaks, WHO has prioritized the development of vaccines suitable for use in an emergency or outbreak scenario. There are more than 40 vaccine candidates being developed using different platforms. Most of them are in the pre-clinical stage of development, but there are a few candidates which are the subject of clinical trials.^11^

Accurate diagnosis is essential to monitor and control the spread of ZIKV infection, as well as to provide guidance for pregnant women, or those planning to have children.^12,13^ Indeed, despite ZIKV infection occurring through the bite of infected mosquitoes of the genus *Aedes*, the virus can also be passed from mother to foetus and through exchange of body fluids.^14–16^ Molecular tests for ZIKV are the most reliable and specific, and there are several diagnostic tools authorized by the United States Food and Drug Administration under Emergency Use Authorization (EUA), while others are included in the WHO Emergency Use Assessment and Listing.^13,17^ However, ZIKV in blood/serum is short-lived (3–5 days), and up to 10 days in urine;^18^ therefore, a serological assay to measure immune exposure by antibody detection is suggested 7 days post onset of symptoms.^13,17^ A major problem with detection of anti-ZIKV antibodies is cross-reactivity with other flaviviruses, such as dengue virus (DENV).^19–21^ Specifically, the envelope glycoprotein E contain regions highly conserved between ZIKV and DENV, which are targets for binding and neutralizing antibodies.^22^ At the time of the outbreak in 2015/16, the only authorized EUA test was the Zika Mac-ELISA (enzyme-linked immunosorbent assay), which addresses the antibody cross-reactivity with other flaviviruses by requiring a 4-fold higher antibody titre in a plaque reduction neutralization assay against ZIKV than other flaviviruses to confirm infection.^17^ Recently, to avoid cross-reactivity, serological assays have been developed targeting antibodies specific to the non-structural protein 1 (NS1), which is secreted by ZIKV-infected cells into the body fluids.^23,24^

Standardized, validated assays are of utmost importance not only for diagnosis of ZIKV infection and surveillance but also for vaccine development as anti-ZIKV antibody responses will most likely be used as immune markers for the evaluation of vaccine efficacy when large clinical trials with immunological/safety endpoints may be possible, but not clinical efficacy endpoints.^25,26^ Standardized assay methods have their limitations, however, due to variations in operator performance, different implementations among laboratories, and a lack of consistency over time as new refinements and technologies are introduced. Instead, the use of a biological standard that is common to all assays allows for calibration and harmonization of the data from different laboratories worldwide and bridges through advances in technology. The WHO International Standards (WHO ISs) are the highest order of reference materials and allow comparison of biological measurements (activity/potency) by defining an internationally agreed unit, the International Unit (IU^27^). In-house or kit working standards should be calibrated using the WHO IS and the results reported in IU. Use of WHO IS has an impact on product development, for example, the majority of the molecular assays for hepatitis C virus (HCV) detection and quantification are calibrated to the WHO IS for HCV RNA;^28^ furthermore, the potency of Yellow Fever vaccines is expressed in IU^29^ by calibration of the assay to the 1st International Standard for Yellow Fever vaccine.^30^

A multi-centre international collaborative study was organized to evaluate whether a reference preparation of sera or plasma from ZIKV-infected individuals could help harmonize serological assays for ZIKV antibody and the main outcomes of the study are presented in this article.

## Results

### Results returned from neutralization assays

Nine laboratories (2, 3, 7, 8, 11, 12, 13, 14, and 15; Table 1) returned data for live virus neutralization assays, and two laboratories (16 and 18) reported results for a reporter virus particle (RVP) neutralization assay. All neutralization assays were performed using a ZIKV Asian lineage, except laboratory 11a, which also included a ZIKV African lineage (MR766).

**Table 1:** Laboratory codes and assay methods

Lab code	Assay method	Analyte (anti-)/ZIKV strain	Readout
1a	In-house indirect ELISA	Whole virus inactivated/Asian strain— PRVABC59	OD
1b	Commercial indirect ELISA (IgG)	NS1/African strain—MR766	ELISA unit
1c	Commercial capture IgM assay (qualitative)	NS1/not disclosed	POS/NEG
2a	Neutralization assay	Envelope protein/Asian strain—French Polynesia (PF13/251013-18)	TCID_50_
2b	Neutralization assay	Envelope protein/Asian strain—French Polynesia (PF13/251013-18)	PRNT_50_^a^
2c	Commercial indirect ELISA (IgG)	NS1/not disclosed	Relative unit/mL
2d	Commercial indirect ELISA (IgM) (qualitative)	NS1/not disclosed	POS/NEG
3	Neutralization assay	Envelope protein/ Foraleza/2015 Brazil/2015	PRNT_50_^b^
4	Commercial indirect ELISA (IgG)	NS1/not disclosed	Relative unit/mL
5a	In-house competitive ELISA	NS1/Asian lineage	OD
5b	In-house indirect ELISA	NS1/Asian lineage	OD
6a	In development—commercial antigen-coated nanoparticle agglutination (qualitative)-IgM	NS1/Suriname strain	POS/NEG
6b	In development—commercial antigen-coated nanoparticle agglutination (qualitative)-IgG	NS1/Suriname strain	POS/NEG
7	Neutralization assay	Envelope protein H/PAN/2015/CDC-259359 (Human/2015/Panama)	PRNT_50_^b^
8	Neutralization assay	Envelope protein PRVABC59	MN_50_^a^
9	Surface plasmon resonance	Envelope protein—Brazil 2016 strain	Resonance units
10	Capture IgM ELISA (qualitative)	Envelope protein—Asian lineage	POS/NEG
11a	Neutralization assay	Envelope—strains MR766 (Rhesus/1947/Uganda)	Relative copy number by RT-qPCR
11b	Neutralization assay	Envelope—Asian lineage	Relative copy number by RT-qPCR
12	Neutralization assay	Envelope—Paraiba/2015	PRNT_50_
13	Neutralization assay	Envelope—PRVABC59	PRNT_50_
14a	Capture IgM ELISA (qualitative)	Multiple antigens/HPF2013	POS/NEG
14b	Capture IgG ELISA (qualitative)	Multiple antigens/HPF2013	POS/NEG
14c	Neutralization assay	Envelope—Asian lineage (HPF2013)	IC_50_^a^
15a	Neutralization assay (day 3 p.i.)	Envelope—PRVABC59	IC_50_
15b	Neutralization assay (day 4 p.i.)	Envelope—PRVABC59	IC_50_
15c	Indirect epitope lockade of binding (qualitative)	NS1/Uganda and Suriname strains	POS/NEG
16	Reporter virus particle neutralization assay	Zika prM/E on a West Nile Virus replicon system	IC_50_^a^
17	Commercial capture IgM/IgG (qualitative)	NS1/not disclosed	POS/NEG
18	Reporter virus particle neutralization assay	Protein C-prM-E from ZIKV strain SPH2015 on reporter virus particles	IC_50_^a^
19	Commercial IgM lateral flow (qualitative)	NS1/not disclosed	POS/NEG

*EIA* enzyme immunoassay, *Neut* neutralization assay, *p.i.* post infection, *PRNT* plaque reduction neutralization test, *RT-qPCR* reverse transcription-quantitative polymerase chain reaction, *TCID50* 50% tissue culture infectious dose, *OD* optical density, *POS/NEG* positive/negative, *MN*_*50*_ 50% microneutralization titre, *IC*_*50*_ half-maximal inhibitory concentration

Note: Labs 2 and 4 used the same commercially available quantitative ELISA; labs 1c and 17 have the same commercially available qualitative ELISA

^a^Calculated as IC_50_ from inhibition curve

^b^Inverse of the dilution, which achieved 50% reduction in plaques

All laboratories identified correctly all the positive samples, these being either the pools of convalescent plasma/serum (samples S14, S48, S61, and S80—Table 2) or immunized trans-chromosomal (Tc) bovine immunoglobulins (samples S1 and S26). All the negative controls, plasma pool (sample S38), serum pool (sample S2), and naive Tc bovine immunoglobulin (sample S6) were negative in all the assays, except for that from lab 15, which indicated that sample S2 was a low positive. Seven out of 14 data sets did not show any cross-reactivity with the DENV reference preparations (samples S53, S79, and S93). Labs 3, 13, and 14c, however, scored either (lab 3) or both (labs 13 and 14c) of the DENV serotypes 1 and 2 (sample S53 and S79) positive; lab 7 identified DENV serotype 2 and 3 (samples S79 and S93) as positives. Labs 8 and 15 reported cross-reactivity with DENV serotype 3. In all cases, the estimated potency of the DENV samples was lower than the ZIKV samples and therefore discriminatory. Table 3 shows the geometric means (GMs) of the results, which were provided as either median 50% plaque reduction neutralization titres (PRNT_50_) or endpoint titre estimates.

**Table 2:** Collaborative study samples

Sample code	Sample description	Donor
S1/TcEprot	Purified human IgG from Tc bovine immunized with plasmid DNA encoding Zika E antigen	Eddie J. Sullivan, SAB Biotherapeutics Inc., USA
S2/HuNeg	Human negative serum	NHSBT
S6/TcNeg	Purified human IgG from naive Tc bovine, negative control	Eddie J. Sullivan, SAB Biotherapeutics Inc., USA
S14/HuUS	Pool of 2 donors ZIKV-positive, plasma SD-extracted	Joseph Mauro, Boca Biolistics, Pompano Beach, FL, USA
S26/TcZIK	Purified human IgG from Tc bovine immunized with whole killed Zika virus	Eddie J. Sullivan, SAB Biotherapeutics Inc., USA
S38/HuNeg	Human plasma negative	NHSBT
S48/HuPR	Pool of sera from 8 Zika-positive donors from Puerto Rico	Barney Graham and Julie Ledgerwood, Vaccine Research Centre, NIAD/NIH, USA
S53/D1	Dengue serotype 1 International Reference Reagent	NIBSC
S61/HuCAR	Pool of sera from 100 Zika-positive donors from the Caribbean SD-extracted	Richard Brindle, CARPHA, Trinidad and Tobago
S79/D2	Dengue serotype 2 International Reference Reagent	NIBSC
S80/cIS	Candidate Zika International Standard (serum)	Ines Ushiro-Lumb, NHSBT, Colindale, UK
S93/D3	Dengue serotype 3 International Reference Reagent	NIBSC

*CARPHA* Caribbean Public Health Agency, *NHSBT* National Health Service Blood and Transplant, *NIH* National Institutes of Health, *SD* solvent detergent, *Tc* = trans-chromosomal

**Table 3:** Neutralization titres, reported by the participants, as the geometric mean of three independent experiments

	Laboratory													
Sample	2a	2b	3	7	8	11a^a^	11b^a^	12	13	14	15a	15b	16^b^	18^b^
S1/TcEprot (+)	509	1724	254	10	619	2528	2001	230	2259	460	308	182	2407	832
S2/HuNeg (−)	<10	ND	<10	<10	<10	Neg	Neg	<5	<10	<40	16	14	<30	ND
S6/TcNeg (−)	<10	ND	<10	<10	<10	Neg	Neg	<5	<10	<40	<10	<10	<30	ND
S14/HuUS (+)	7356	6062	4064	178	5812	317,650	19,610	2299	28,298	4268	3810	1726	3700	6709
S26/TcZIK (+)	162	697	320	10	282	674	614	289	1069	276	164	65	1976	324
S38/HuNeg (−)	<10	ND	<10	<10	<10	Neg	Neg	<5	<10	<40	<10	<10	<30	ND
S48/HuPR (+)	2436	2714	1613	46	4975	346,681	10,358	1306	7833	4887	690	387	6818	4105
S53/D1 (−)	<10	ND	18	<10	<10	Neg	Neg	<5	27	41	<10	<10	<30	ND
S61/HuCAR (+)	4299	2003	453	100	3114	88,555	2404	488	2801	1572	416	487	3515	3020
S79/D2 (−)	<10	ND	<10	10	<10	Neg	Neg	<5	27	43	<10	<10	<30	ND
S80/cIS (+)	4467	1477	640	68	2641	23,002	5443	939	7443	1094	725	951	3796	2590
S93/D3 (−)	<10	ND	<10	10	12	Neg	Neg	<5	<10	<40	16	20	<30	ND

*Neg* negative; *ND* not determined; expected status of each sample is indicated in the sample name as ZIKV antibody positive (+) or negative (−)

^a^Lab 11 detection method is ZIKV relative copy number by RT-qPCR, with lab 11a using an African isolate and 11b an Asian lineage ZIKV

^b^Reporter virus particles neutralization assay

### Harmonization of the neutralization titres by the candidate IS

**Fig. 1:**
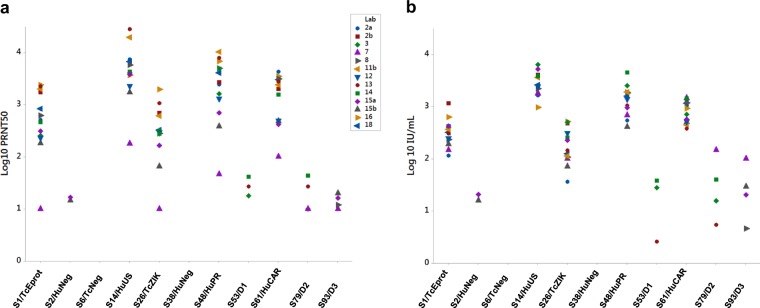
Harmonization of the samples’ potency when reported as relative to the candidate International Standard. **a** The geometric mean of neutralization titres for each sample as reported by the participants following three independent experiments (Table 3) are plotted; **b** neutralization titres were calculated relative to the candidate International Standard (sample S80), assuming an arbitrary value of 1000 IU/mL (Table 4). Samples S2, S6, and S38 are the negative controls; samples S53, S79, and S93 are the anti-dengue serum samples

Neutralization titres provided by the participant laboratories differed more than 100-fold for some samples (Table 3, Fig. 1a). Variability across neutralization assays is intrinsic of this type of assay, where multiple factors play a role such as target cells, virus stock, timings, and other reagents used. Specifically, the different assay readouts, and their different ranges, have a critical impact; for example, lab 11 detected ZIKV infection by a reverse transcription-quantitative polymerase chain reaction (RT-qPCR) readout and has the highest titre values. To evaluate the effect of normalizing the data using a standard preparation, the results in Table 3 were expressed as relative to the values of sample S80 within each assay (Table 4). Sample S80, a pool of sera from Zika, confirmed cases returning to the UK, provided by the National Health Service Blood and Transplant (NHSBT) was chosen as the candidate International Standard because it was donated in a large quantity sufficient for the production of over 2000 ampoules and the material had not undergone any treatments prior to freeze-drying at National Institutes for Biologicals Standards and Control (NIBSC). Expression of the potency of each sample as relative to sample S80 reduced the variation between the labs for each positive sample (Table 5, Fig. 1b). In Table 5, the GM of each sample was calculated using the neutralization data from all labs except for lab 11a, where the potency values provided for each sample were considered outliers in comparison with the remaining data set. Since this was the only assay using ZIKV of African lineage, it was not possible in this study to determine whether the higher neutralization titres were lineage- or assay-dependent.

**Table 4:** Neutralization titres expressed relative to sample 80, assuming an arbitrary value of 1000 IU/mL

	Laboratory													
Sample	2a	2b	3	7	8	11a^a^	11b^a^	12	13	14	15a	15b	16^b^	18^b^
S1/TcEprot (+)	114	1167	397	147	234	166	368	244	304	421	425	191	634	321
S2/HuNeg (−)	–	–	–	–	–	–	–	–	–	–	21	16	–	–
6/TcNeg (−)	–	–	–	–	–	–	–	–	–	–	–	–	–	–
S14/HuUS (+)	1647	4104	6350	1778	2201	15,566	3602	2448	3802	3902	5253	1815	975	2590
S26/TcZIK (+)	36	427	500	100	107	31	113	308	144	253	226	71	521	125
S38/HuNeg (−)	–	–	–	–	–	–	–	–	–	–	–	–	–	–
S48/HuPR (+)	545	1838	2520	681	1884	6874	1903	1391	1052	4468	952	406	1796	1585
S53/D1 (−)	–	–	28	–	–	–	–	–	3	38	–	–	–	–
S61/HuCAR (+)	962	1356	707	1468	1179	3240	442	520	376	1437	547	512	926	1166
S79/D2 (−)	–	–	–	147	–	–	–	–	5	40	–	–	–	–
S93/D3 (−)	–	–	–	100	5	–	–	–	–	–	20	29	–	–

–:Negative or under detection limit of the assay; expected status of each sample is indicated in the sample name as ZIKV antibody positive (+) or negative (−)

^a^Lab 11 detection method is ZIKV relative copy number by RT-qPCR, with lab 11a using an African isolate and lab 11b an Asian lineage ZIKV

^b^Reporter virus particles neutralization assay

**Table 5:** Reduction in the inter-laboratory variance by expressing the neutralization titres as relative to the candidate International Standard

	GM		GCV	
	NT	IU/mL	Raw data	Relative to sample 80
S1/ TcEprot (+)	481	318	337%	84%
S2/HuNeg (−)	15	18		
S6/TcNeg (−)				
S14/HuUS (+)	4290	2756	240%	70%
S26/TcZIK (+)	268	173	274%	126%
S38/HuNeg (−)				
S48/HuPR (+)	2023	1339	343%	93%
S53/D1 (−)	27	14	52%	338%
S61/HuCAR (+)	1222	808	217%	62%
S79/D2 (−)	23	32	108%	327%
S80/cIS (+)	1511	1000^a^	243%	
S93/D3 (−)	14	23	35%	257%

Geometric mean value of the neutralization titres (NT) as reported by the participants or expressed as relative (IU/mL) to sample S80 with an assigned potency of 1000 IU/mL; expected status of each sample is indicated in the sample name as ZIKV antibody positive (+) or negative (−)

^a^Assigned value

### Anti-ZIKV IgM detection in the pools of plasma/sera

Seven laboratories (1, 2, 6, 10, 14, 17, and 19—Table 1) processed the panel of samples in qualitative enzymatic immunoassays for the detection of anti-ZIKV immunoglobulin M (IgM). Most of the laboratories were able to detect specific IgM in known positive human convalescent samples S14, S48, S61, and S80. No signal was detected in the negative pools of plasma (sample S38) and sera (sample S2), or in the dengue reference preparations (samples S53, S79, and S93). The Tc bovine antibodies (samples S1, S6, and S26) were all negative as well. This was expected because the transgenic bovines do not generate human IgM.^31^ Laboratory 2d found all the samples negative. Laboratory 6a scored positive the Tc bovine preparations and scored negative sample S80 (Supplementary Table 1). This result was due to the assay being under development at the time of this study, and an inefficient blocking method being used, which has now been improved to resolve these issues (personal communication with the study participant).

### Performance of the sample panel in other assays

Anti-ZIKV binding immunoglobulin G (IgG) antibodies were detected in qualitative (assays 6b, 14b and 15a, Table 1) and quantitative enzymatic assays (1a, 1b, 2c, 4, 5a and 5c, Table 1). Qualitative methods correctly identified all the positive samples except for the Tc bovine immunoglobulin (sample S1) from an animal immunized with plasmid DNA coding the E protein. Lab 14 only was able to detect this as a positive sample: this assay included multiple ZIKV antigens, while lab 6 and 15 assays are NS1-specific. All the control samples were correctly scored as negative, and no cross-reactivity was reported with the dengue samples (Supplementary Table 1).

Most of the quantitative ELISAs are specific for ZIKV NS1 and show no cross-reactivity with the dengue samples except for lab assay 1a, which is an in-house assay using whole ZIKV as coating antigen. As expected, this assay was the only quantitative ELISA able to record a positive result for sample 1, as this sample is derived from the Tc bovine immunized against ZIKV E protein only.

**Fig. 2:**
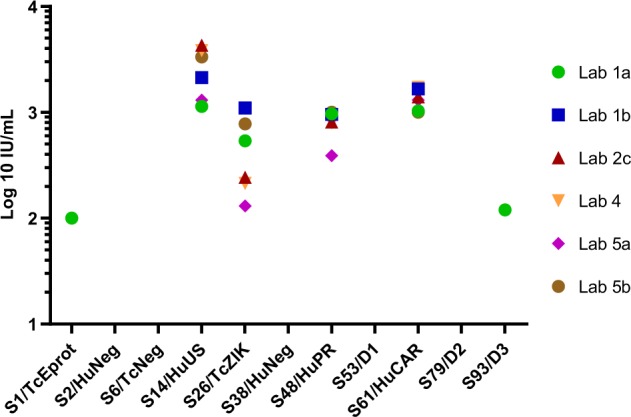
ELISA potencies calculated relative to the candidate International Standard. The samples were analysed using commercial and in-house quantitative assays as described in Table 6. The potencies in IU/mL were calculated from the geometric mean of three independent assays of the data expressed relative to the value of sample S80, which was assigned an arbitrary value of 1000 IU/mL Only the estimates calculated for labs 2c and 4 were valid by parallel line analysis

The data returned were reported using different readouts (Table 6) making comparisons between different ELISAs difficult; however, by reporting the results as a potency relative to sample S80, in a similar way to that used for the neutralization data, there was a good level of agreement between laboratories (Fig. 2). However, only labs 2 and 4 produced a data set that allowed for the calculation of a relative potency by parallel line analysis.

**Table 6:** Quantitative ELISA data

	1a^a^	1b^b^	2c^b^	4^b^	5a^c^	5b^c^
S1/TcEprot (+)	0.1	–	–	–	–	–
S2/HuNeg (−)	–	–	–	–	–	–
S6/TcNeg (−)	–	–	–	–	–	–
S14/HuUS (+)	1.1	58.4	1365.0	1048.1	10.0	10.0
S26/TcZIK (+)	0.5	30.0	77.2	58.2	1.0	2.3
S38/HuNeg (−)	–	–	–		–	–
S48/HuPR (+)	1.0	26.2	257.8	218.0	3.0	3.0
S53/D1 (−)	–	N/T	–		–	–
S61/HuCAR (+)	1.0	45.6	446.5	472.4	10.0	3.0
S79/D2 (−)	–	N/T	–		–	–
S80/cIS (+)	1.0	27.3	318.1	273.6	7.7	3.0
S93/D3 (−)	0.1	N/T	–	–	–	–

N/T = not tested; expected status of each sample is indicated in the sample name as ZIKV antibody positive ( + ) or negative (−)

^a^In-house assay, potency relative to internal standard

^b^Commercial assay, ELISA relative unit based on kit standard—labs 2c and 4 used the same kit

^c^In-house assay, inverse of the last positive dilution

Lab 9 analysed the panel of samples by surface plasmon resonance (SPR) targeting the E protein of ZIKV (Brazil 2016 isolate). The samples could be ranked similarly to their neutralization titres (Table 5) with the pools of sera/plasma producing higher resonance units (RU) than the human Tc bovine antibodies (comprising individual animals). All negative samples were below the cut-off limit, while dengue preparations scored above the cut-off limit of 30 RU, but at least five times lower than the weakest ZIKV-positive sample (sample S1, Supplementary Table 2).

### Stability study

**Fig. 3:**
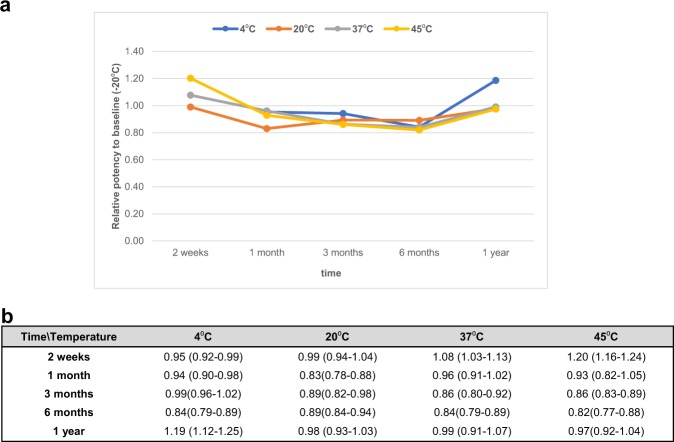
Thermal degradation assessment of the candidate International Standard for ZIKV antibody. Freeze-dried ampoules of sample S80 (NIBSC code 16/352) were stored at five different temperatures (45, 37, 20, 4, and −20 °C). At each time point, three vials were retrieved and reconstituted with 0.25 mL of molecular grade water. Each vial was assessed in triplicate in the in-house ELISA. Data are reported relative to the storage temperature −20 °C; **a** the graph shows the variation in potency against time; **b** the bottom table contains the mean value of three independent experiments of the relative potency with the 95% confidence limit within parentheses

The stability of the candidate IS (sample S80) was assessed by accelerated thermal degradation testing. Ampoules were placed into storage at 45, 37, 20, 4, and −20 °C, and retrieved at the following time points: 2 weeks, 1 month, 3 months, 6 months, and 1 year and stored at −20 °C until assayed. The freeze-dried preparations were reconstituted as per instruction for use and tested concurrently in triplicate by in-house ELISA. Data are reported as relative to the baseline temperature −20 °C (Fig. 3). The relative potency after 1 year at each temperature did not differ from the baseline (e.g., 1 year at 45 °C relative potency 0.97), suggesting that the material is sufficiently stable for long-term storage and can be shipped at ambient temperature.

## Discussion

The purpose of an IS is to harmonize data produced by laboratories worldwide.^27^ The concept of WHO ISs is based on the fact that laboratories use their own assays rather than one single assay. This means that the WHO ISs usually satisfy needs of users who are conducting different kind of assays. One of the objectives of a collaborative study such as this one is to demonstrate the suitability of the IS in the assays used in the collaborative study.

Through such multi-centre collaborative study, we have evaluated the ability of a pool of sera from ZIKV-infected patients to reduce the variability of the results from neutralization assays between 11 laboratories worldwide. Data returned from 14 methods showed a large magnitude of titres, which likely reflects the different methodologies used rather than performance of the assays (Table 3). All the assays were able to correctly identify the positive samples, and only one laboratory identified the negative serum preparation (sample S2) as a false positive. Some assays showed cross-reactivity with the Dengue reference preparations in the panel; however, this may reflect the presence of cross-neutralizing antibody epitopes in the ZIKV E protein.^22^ Indeed, there was no cross-reactivity observed in those binding assays targeting NS1 protein only (Table 6).

Neutralization titres of the collaborative study samples as reported by the participants differed by up to 100-fold; however, normalization by the candidate IS (sample S80) produced more comparable results with a reduction of inter-laboratory variance (Tables 4, 5 and Fig. 1). Similar results have been obtained in the collaborative study for the establishment of the 1st WHO IS for Ebola antibody,^32^ but inter-lab variability is often specific to the assay target and such comparisons are not easily made. Interestingly, the only method using ZIKV of African lineage produced the highest neutralization titres, about 10 times higher than the other assays (Table 4). As only one laboratory performed the neutralization assay with an African isolate, using a detection method (RT-qPCR) not comparable with other neutralization assays in this collaborative study, no conclusions can be inferred from these data. Although the ZIKV African lineage is more virulent than the Asian lineage in vitro and in animal models,^33–36^ a previous study has not reported a difference in neutralizing antibodies between the two lineages in convalescent human serum or plasma.^37^ Further investigations are clearly needed to resolve this issue so as to understand the possible reasons for the difference in titres.

The candidate IS and the panel of samples were further characterized in other serological methods such as ELISA and SPR, where the samples ranked similarly to the neutralization results. Importantly, expressing the ELISA results as relative to sample S80 allowed the comparison of results from different kits and in-house methods (Table 6 and Fig. 2). Unfortunately, it was not possible to assign the candidate IS as a calibrator for ELISA methods from this collaborative study. Only two sets of data from labs 2 and 4 were statistically valid by parallel line analysis (see methods), and therefore it was not possible to demonstrate that the candidate IS was able to reduce inter-laboratory variance. Suitability of the IS for anti-ZIKV antibodies in ELISA methods would require an additional collaborative study with a sufficient number of laboratories conducting quantitative ELISA and fulfilling statistical criteria for this kind of study.

It is important to highlight that all pools of convalescent serum/plasma in this study had some reactivity to DENV. Anti-DENV reference materials from NIBSC (samples S53, S79, and S93) were included in the study to assess assay specificity. Although cross-reactivity against DENV of neutralizing antibodies elicited by ZIKV infection has been reported, the lower titre (4–10-fold) allows for discrimination of the ZIKV infection in a DENV-immune patient.^38^ In this study, all the assays were able to differentiate ZIKV antibodies in the samples provided, confirming that the candidate International Standard (sample S80) contains ZIKV-specific antibodies. However, due to the presence of cross-reactive anti-DENV antibodies, the IS for anti-ZIKV antibodies cannot be used for the validation of specificity of ZIKV serological assays.

The outcome of this study was that the NHSBT pool of convalescent serum sample (Sample Code S80) was established by the WHO Expert Committee on Biological Standardization in October 2018 as the 1st WHO IS for anti-Asian lineage ZIKV antibody to be used in the standardization and assessment of neutralization assays with an assigned unitage of 250 IU per ampoule.^39^ The IS is available from the NIBSC catalogue (www.nibsc.org/), code 16/352, in ampoule numbers anticipated to be available for 5–10 years; stability studies have shown that the preparation is also stable for long-term storage. Furthermore, sample S14, the pool of convalescent plasma (S14) from Boca Biolistics, is also available in the NIBSC on-line catalogue (code 16/320) as a working reagent calibrated to the 1st WHO IS in this collaborative study. The calculated value of 16/320 is 2756 IU/mL, with 95% confidence limits of 2003 to 3792.

This study has shown how normalization of the data from different assays by the IS allows for comparison and harmonization of results from laboratories worldwide. The use of the IS reduced the inter-laboratory variation, albeit there is still up to a 10-fold difference in the titres reported in IU/mL. Use of standardized methods with a validated standard operation procedure and sharing of the same reagents could be a further step to reduce inter-laboratory variations; however, this is beyond the scope of this work and could be evaluated by the scientific community and regulatory bodies working on Zika. Nevertheless, calibration of assays using the IS for ZIKV antibodies will allow for the consistency of antibody measurements among laboratories, the comparative analysis of candidate vaccines in clinical trials, which aim to determine protective antibody levels in human or animal models, leading to a batch/lot release specification; therefore, resulting in expedited licensing and subsequent availability of vaccines.

In conclusion, WHO standardization activities led by NIBSC resulted in the development and establishment of the 1st IS for anti-Asian lineage ZIKV antibody. This is an important achievement for the scientific community across ZIKV diagnostics, prevention, and treatment. It is expected that the above-mentioned standard will be used in the clinical development of ZIKV vaccines with the aim of reporting results of the assessment of the immune response in IUs. There have been examples of missed opportunities in using the ISs for development of new vaccines. There are several reasons, but the most important are: (1) lack of understanding among principal investigators of the benefits that the IS provide in the interpretation of data from clinical trials and (2) misunderstandings about assay standardization that less experienced vaccine developers are facing when establishing testing procedures for vaccines under development. Multiple vaccine candidates have shown robust protection against ZIKV challenge in animal models, but the demonstration of the protection in humans remains a challenging task. For the comparability of clinical trial results, it is critical to use WHO IS as a basis for assay standardization and optimization. Correct interpretation of the results from clinical trials is one of the essential elements to assure quality, safety, and efficacy of vaccines and diagnostics. Feedback from users will help WHO and its Collaborating Centre, NIBSC, to advance the further use of this standard as well as the development of other standards and reagents that may improve standardization of assays used in the clinical evaluation of Zika vaccines.

## Methods

### Participants

Nineteen participants with an anti-ZIKV antibody assay in use in their laboratories completed the study. Prerequisites for participation in the collaborative study are: (1) availability of reliable assay for detection of Zika antibody and (2) willingness to participate. This approach provides a platform to evaluate the suitability of the standard to harmonize as wide a range of assay types as possible, which is essential for robustness of WHO standards. The participants were from six countries: Germany (1), UK (2), Korea (1), Trinidad and Tobago (1), Denmark (1), and USA (13). All laboratories are referred to by a code number allocated at random and are not reflected in the order of listing at the end of the paper. Participating labs included 11 government research, public health, medical counter-measure, and regulatory organizations; three university and research organizations; five developers of biologics, assays, and reagents.

### Samples

The project was approved by the NIBSC Human Material Advisory Committee (project 16/005MP). Plasma and sera were donated to NIBSC anonymized by the organizations listed in Table 2. All patients signed an informed consent to the use of their sera/plasma.

Collaborative study samples were provided to the participants blind coded and are listed in Table 2. Four pooled samples of plasma (sample S14) or serum (samples S48, S61 and S80) from individuals who were designated as having been infected by ZIKV were prepared at NIBSC, filled in 0.25 mL/ampoule, freeze-dried and stored at −20 °C. Prior to filling, two pools, sample S14 and S61, were treated with solvent and detergent^40^ to inactivate blood-borne viruses, as on initial screening these were found to be positive for HIV RNA, HBV antigen or HCV RNA. Post treatment testing for the presence of RNA was negative.

Additional samples were human anti-ZIKV IgG antibodies purified from plasma collected from Tc cattle^31^ immunized with ZIKV immunogens comprising either inactivated virus (Puerto Rico strain cultured in C6/36 insect cells, sample S26), plasmid DNA coding full-length ZIKV E protein (pWRG/Zika PrM-E, sample S1), or the naive control (sample S6).^41^ Upon receipt at NIBSC, the purified Tc bovine IgG samples were diluted to a target protein concentration of 2.5 mg/mL in sterile phosphate-buffered saline (PBS)/Ca^2+/^Mg^2+^ supplemented with 5% human serum albumin and aliquoted into 0.1 mL volumes and stored at −20 °C.

Negative human plasma and serum were from single donation packs from the UK; all packs were tested for blood borne markers (HIV, HCV, hepatitis B surface antigen and syphilis) and found to be negative.

Anti-DENV serotype antibodies (1–3; serotype 4 was unavailable) were sourced from NIBSC.^42^

### Collaborative study assay methods

Assays used by participants are summarized in Table 1. Where laboratories performed multiple assay methods, laboratory codes are suffixed by a letter indicating the different methods, for example, labs 11a and 11b. The two main assay methods were neutralization of ZIKV, with two laboratories using a RVP system instead of the wild-type virus, and enzyme immunoassays. Anti-ZIKV IgM responses were detected by qualitative assays only, while anti-ZIKV IgG assays were both quantitative and qualitative. SPR was also performed.

### NIBSC in-house ELISA

Nunc Maxisorp 96-well plates were coated overnight at 4 °C with ZIKV (Asian strain—PRVABC59)-containing supernatant from VERO cells diluted in PBS. Conditioned media from uninfected VERO cells was used as a negative control. Virus was fixed with 0.1 mL per well of 4% paraformaldehyde diluted in PBS for 30 min at room temperature. Plates were washed three times with PBS, and then blocked with 0.2 mL of 10% foetal bovine serum in PBS for 1 h at room temperature. Plates were washed three times with PBS containing 0.05% Tween-20 (PBST). A measure of 0.1 mL of serum samples diluted 1/400 in blocking buffer were added to the plates in triplicate and incubated at room temperature for 1 hour. Wells were washed with PBST and 0.1 mL of anti-human horse radish peroxidase conjugate antibody (Jackson ImmunoResearch Inc., cat. no. 109-035-088) diluted 1:5000 in blocking buffer were added to each well. After 1 h incubation at room temperature, plates were washed with PBST and developed by adding 3,3′,5,5′-Tetramethylbenzidine Substrate Systems (Neogen Europe, Ltd.). Reactions were stopped after 10 min by the addition of 1 M H_2_SO_4_. For the analysis, absorbance from each well was subtracted from the average of absorbance of the diluent-only wells. An optical density >0.1 after subtraction with the correspondent negative control was arbitrarily chosen as a positive result.

### Study plan

Participants were requested to test the samples using the method(s) in use in their laboratories for the detection of antibodies to ZIKV. Participants were asked to perform three independent assays on different days. An Excel reporting sheet was provided with suggested dilutions for assaying each study sample. For each assay, participants were requested to make at least two independent series of dilutions of the study samples and assay all samples concurrently if feasible.

### Statistical analysis

For the neutralization assays, the GM of the potency of each sample was calculated from the endpoint titres or PRNT_50_ provided by the participants. GMs were calculated only when more than half of the results for a sample produced a positive response.

Quantitative ELISA data were analysed using a parallel line model with untransformed or log-transformed responses. Calculations were performed using the European Directorate for the Quality of Medicines & Healthcare (EDQM) software CombiStats™.^43^ Model fit was assessed visually, and non-parallelism was assessed by calculation of the ratio of fitted slopes for the test and reference samples under consideration. The samples were concluded to be non-parallel when the slope ratio was outside the range 0.80–1.25. Relative potency estimates from all valid assays were combined to generate an unweighted GM for each laboratory and assay type, with these laboratory means being used to calculate overall unweighted GMs for each analyte.

Variability between laboratories has been expressed using geometric coefficients of variation (GCV = [10^s^ − 1] × 100%, where *s* is the standard deviation of the log_10_-transformed estimates). Further assessment of agreement in GM results for each pair of laboratories was performed by calculating Lin’s concordance correlation coefficient with log-transformed data, although these values are only based on a small number of samples (between five and seven in all cases). Calculations were performed using the R package DescTools.^44^

### Reporting summary

Further information on research design is available in the Nature Research Reporting Summary linked to this article.

## Supplementary information

Supplementary Tables

Reporting Summary

## Data Availability

Raw data can be made available upon request, but the participant’s name will be anonymized. The study samples can also be made available, until depletion of stocks.
