# Supplementary material for: Harmonization of Zika neutralization assays by using the WHO International Standard for anti-Zika virus antibody
Source: NPJ Vaccines. 2019 Oct 14;4:42. doi: 10.1038/s41541-019-0135-3 (PMC6791859; doi:10.1038/s41541-019-0135-3)
Supplement: Supplementary file 1 — Supplementary Tables [file 41541_2019_135_MOESM1_ESM.pdf]

Supplementary Table 1 Binding of anti-ZIKV antibodies in the study samples by qualitative ELISAs

The table shows the proportion of qualitative assay run in each lab that returned a positive result for that sample, i.e. 0/3 is no positives out of 3 assays; nt: not tested.

	Laboratory								
	IgM						IgG		
**Sample**	**1c**	**6a**	**10**	**14a**	**17**	**19**	**6b**	**14b**	**15**
S1/TcEprot	0/3	3/3	0/3	0/2	0/3	0/3	0/3	2/2	0/3
S2/HuNeg	0/3	0/2	0/3	0/2	0/3	0/3	0/3	0/2	0/3
S6/TcNeg	0/3	3/3	0/3	0/2	0/3	0/3	0/3	0/2	0/3
S14/HuUS	3/3	2/2	3/3	2/2	3/3	3/3	3/3	2/2	3/3
S26/TcZIK	0/3	3/3	0/3	0/2	0/3	0/3	3/3	2/2	3/3
S38/HuNeg	0/2	0/3	0/3	0/1	0/3	0/3	0/3	0/2	0/3
S48/HuPR	3/3	3/3	3/3	2/2	3/3	3/3	3/3	2/2	3/3
S53/D1	0/3	nt	0/3	0/2	0/3	0/3	nt	0/2	0/3
S61/HuCAR	2/2	3/3	3/3	2/2	3/3	3/3	3/3	2/2	3/3
S79/D2	0/3	nt	0/3	0/2	0/3	0/3	nt	0/2	0/3
S80/cIS	3/3	0/1	3/3	2/2	3/3	3/3	3/3	2/2	3/3
S93/D3	0/3	nt	0/3	0/2	0/3	0/3	nt	0/2	0/3

Supplementary Table 2 Samples analysis by surface Plasmon Resonance

SPR resonance unit are calculated as geometric mean of 3 independent experiments ± standard deviation. Values <20 are consider Negative. Values <30 but >20 are ambiguous; expected status of each sample is indicated in the sample name as ZIKV antibody-positive (+) or negative (-)

**sample**	**Mean ± STDEV**	**Values relative to sample S80**
S1/TcEprot (+)	381.68±9.43	0.12
S2/HuNeg (-)	25±0.16	0.01
S6/TcNeg (-)	4.79±1.22	NEG
S14/HuUS (+)	3821.75±49.33	1.23
S26/TcZIK (+)	446.04±10.72	0.14
S38/HuNeg (-)	13.22±2.12	NEG
S48/HuPR (+)	2447.56±86.41	0.79
S53/D1 (-)	45.43±1.84	0.01
S61/HuCAR (+)	1645.12±22.38	0.53
S79/D2 (-)	76.12±1.55	0.02
S80/cIS (+)	3115.38±113.62	1

S93/D3 (-)	72.75±1.73	0.02
